# Chronic exposure to aflatoxin B1, ochratoxin A, and citrinin in women and children and associated health risk characterization: blood biomonitoring evidence from rural Bangladesh

**DOI:** 10.1186/s12940-026-01330-7

**Published:** 2026-09-02

**Authors:** Nicholas N. A. Kyei, Benedikt Cramer, Hans-Ulrich Humpf, Michael Kuhn, Shafinaz Sobhan, Jannik Veerkamp, Sabine Gabrysch

**Affiliations:** 1 Institute of Public Health, Charité – Universitätsmedizin Berlin, Corporate Member of Freie Universität Berlin and Humboldt-Universität Zu Berlin, Charitéplatz 1, 10117 Berlin, Germany; 2 Faculty of Medicine and University Hospital, Heidelberg Institute of Global Health, Heidelberg University, Im Neuenheimer Feld 324, 69120 Heidelberg, Germany; 3 Potsdam Institute for Climate Impact Research (PIK), Member of the Leibniz Association, P.O. Box 60 12 03, 14412 Potsdam, Germany; 4 Institute of Food Chemistry, Universität Münster, Corrensstr. 45, 48149 Münster, Germany

**Keywords:** Mycotoxins, Biomonitoring, Blood Analysis, Ochratoxin A, Citrinin, Aflatoxin B1, Bangladesh, Risk Characterization

## Abstract

Dietary exposure to mycotoxins—including aflatoxins (AFs), ochratoxin A (OTA), and citrinin (CIT)—represents an important health risk, particularly in low-resource settings with widespread food contamination. This study investigates mycotoxin exposure among 719 participants (433 women and 286 children) of a cluster-randomized trial in rural Bangladesh, using residual blood samples collected during the 2019 endline survey. A total of 712 whole blood and 578 serum samples were analyzed by ultra-high-performance liquid chromatography-tandem mass spectrometry (UHPLC-MS/MS). Serum aflatoxin B1 (AFB_1_)-lysine adducts were quantified following enzymatic digestion and solid-phase extraction. To evaluate potential health risks, we estimated probable daily intake (PDI) from blood concentrations of frequently detected mycotoxins. All whole blood samples contained at least two mycotoxins, and 84% of participants were co-exposed to at least three mycotoxins. Biomarkers for the main blood-relevant mycotoxins were frequently detected, with OTA detected in 100% of whole blood samples and its isomer, 2′R-ochratoxin A (2′R-OTA), detected in 98%, CIT in 91%, and Enniatin B (EnB) in 92%, while AFB_1_-lysine—a biomarker of chronic exposure to the potent carcinogen AFB_1_—was detected in nearly one-quarter of women and 4% of children with serum samples. Beauvericin was detected in 6% of samples. The PDI of OTA exceeded health-based guidance values in 80% of women and 99% of children, while the PDI of CIT was of concern for 12% of women and 27% of children. These findings demonstrate widespread co-exposure to multiple mycotoxins, with OTA, CIT, and AFB_1_ posing notable risks, underscoring the urgent need for targeted interventions to protect vulnerable populations.

**Supplementary Information:**

The online version contains supplementary material available at https://doi.org/10.1186/s12940-026-01330-7.

## Introduction

Mycotoxins are toxic secondary metabolites produced by filamentous molds that frequently contaminate food crops and animal feed under favorable environmental conditions, particularly in a warm and humid climate [15, 18]. Chronic dietary exposure to mycotoxins remains a major public health concern, especially in low-resource settings where food safety regulations and storage infrastructure are often inadequate [37, 39, 46]. Depending on the toxin, prolonged exposure has been linked to a wide range of adverse health effects, including carcinogenicity, nephrotoxicity, immunotoxicity, and developmental toxicity [48, 49, 56, 66, 71, 79].

Human exposure to mycotoxins can be assessed either through food monitoring or biomonitoring. While food monitoring provides valuable occurrence data, it is often limited by heterogeneous contamination patterns, modified toxin forms, and difficulties in obtaining representative samples, particularly in subsistence farming systems [68]. Biomonitoring overcomes many of these limitations by measuring internal exposure biomarkers in biological matrices such as urine and blood, thereby integrating exposure across multiple routes, including dietary intake, inhalation, and dermal absorption [13, 27]. Recent advances in Liquid Chromatography-Tandem Mass Spectrometry (LC–MS/MS) methods have substantially improved the sensitivity and breadth of multi-mycotoxin biomonitoring [1, 29, 51, 70].

Bangladesh’s tropical monsoon climate provides favorable conditions for fungal growth and contamination of staple food crops, making dietary mycotoxin exposure highly relevant [37, 78]. Previous biomonitoring studies in Bangladesh have demonstrated widespread exposure to multiple mycotoxins, particularly ochratoxin A (OTA), citrinin (CIT), deoxynivalenol (DON), aflatoxins (AFs), fumonisins, and zearalenone (ZEN) [2–9]. However, most of these studies relied on urinary biomarkers, which primarily reflect short-term exposure.

Blood and urine biomonitoring provide complementary information on mycotoxin exposure rather than being interchangeable measures of systemic exposure. Urine is the preferred matrix for assessing exposure to polar, rapidly excreted mycotoxins, thereby capturing recent, transient dietary intake. In contrast, blood-based biomarkers are limited to a few compounds which are sufficiently persistent and bioavailable to be detected in circulation, primarily aflatoxin B1-lysine (AFB_1_-lysine), OTA, CIT and Dihydrocitrinone (DH-CIT), as well as some emerging toxins, i.e., those only recently recognized as potential risks, such as enniatin B (EnB) and beauvericin (BEA) [27].

The present study focuses on blood-based biomarkers that provide complementary information to urine biomonitoring on longer-term internal exposure. Among these, OTA is one of the most relevant foodborne mycotoxins due to its long plasma half-life and strong protein binding, making it a robust biomarker of chronic exposure [30]. OTA has been classified by the International Agency for Research on Cancer (IARC) as a Group 2B possible human carcinogen and has been associated with nephrotoxicity, immunotoxicity, and developmental toxicity [36, 49, 64]. CIT, another nephrotoxic mycotoxin frequently co-occurring with OTA, has gained increasing attention because of its potential additive or synergistic renal toxicity. Its main metabolite, dihydrocitrinone (DH-CIT), serves as an important biomarker of CIT biotransformation and exposure [20, 38, 49, 66].

In addition, AFB_1_-lysine, the major serum albumin adduct of aflatoxin B1, is widely recognized as a validated biomarker of long-term aflatoxin exposure and has strong toxicological relevance, given that aflatoxin B1 is classified as a Group 1 human carcinogen by the IARC [49, 64, 66]. Emerging mycotoxins such as EnB and BEA were also included because of growing evidence of cytotoxic, mitochondrial, and endocrine-disrupting effects, despite the absence of formal regulatory limits or full risk assessments by the European Food Safety Authority (EFSA) or Joint FAO/WHO Expert Committee on Food Additives [14, 41]. Finally, we included 2’R-ochratoxin A (2’R-OTA), a diastereomer of OTA formed during thermal processing of contaminated food products, due to its increasing recognition as a marker of processed-food exposure and its potential toxicological relevance [47, 75, 77].

This study builds on our previous urinary biomonitoring investigation conducted in the same rural Bangladeshi cohort [52], which identified high occurrence and co-occurrence of OTA and CIT among pregnant women. Here, we analyzed blood samples from women and young children to determine whether these co-exposure patterns reflect sustained, longer-term exposure, to characterize child exposure in this cohort for the first time, and to capture mycotoxins that are better suited to blood-based than urinary detection. Samples were screened for a broad panel of mycotoxins and metabolites (Table S1), with particular focus on AFB_1_-lysine, OTA, CIT, DH-CIT, EnB, and BEA due to their toxicological relevance, biomarker suitability, and/or expected occurrence; other analytes, including DH-CIT, BEA, 2’R-OTA, and several qualitatively monitored compounds, were also explored. The specific objectives were to (i) characterize the occurrence and co-exposure patterns of multiple mycotoxins in women and young children in rural Bangladesh and (ii) estimate the probable daily intake (PDI) of the most frequently detected mycotoxins to assess dietary exposure levels of public health concern in this population.

## Materials and methods

### Study population

The present study uses residual blood samples collected during the 2019 endline survey of the *Food and Agricultural Approaches to Reducing Malnutrition* (FAARM) cluster-randomized controlled trial, conducted in two rural sub-districts of Habiganj district, Sylhet division, Bangladesh from 2015 to 2020 (ClinicalTrials.gov ID: NCT02505711). No trial-related procedures were undertaken as part of the present analysis. Habiganj is a predominantly rural, agrarian district in Sylhet division, northeastern Bangladesh, characterized by smallholder and subsistence farming, a tropical monsoon climate, seasonal flooding in low-lying lands, and limited formal food safety infrastructure [82]. The FAARM trial enrolled 2705 married women from 96 settlements who reported being 30 years of age or younger, having access to at least 40 m2 of land, and declared an interest in gardening. The trial assessed the impact of a homestead food production program (HFP), implemented by the nonprofit Helen Keller International, on maternal and child undernutrition. Further details on the FAARM trial design are available in the published study protocol [82]. As an extension of FAARM, the *Maternal Exposure to Mycotoxins and Adverse Pregnancy Outcomes* (MEMAPO) study, a nested prospective cohort study with urinary biomonitoring, was conducted from 2018 to 2020. MEMAPO followed 439 pregnant FAARM participants from early pregnancy to investigate the effects of maternal mycotoxin exposure on adverse pregnancy outcomes [54]. Another sub-study of FAARM, the *Food Hygiene to reduce Environmental Enteric Dysfunction* (FHEED) study, was designed to analyze the impact of the combined HFP and food hygiene intervention on household food hygiene practices, contamination of complementary foods, and intestinal infection and inflammation among children 0 to 24 months old [60].

The present analysis draws on participants from these two nested FAARM sub-studies. Maternal analysis is based primarily on women enrolled in MEMAPO, whereas child analysis includes children from both MEMAPO and FHEED. The analysis is restricted to women and children with sufficient residual blood volume available after hematological and micronutrient testing (see *Biosample collection*, below); it therefore does not constitute a random sample of the full FAARM cohort of 2705 women, nor is it representative of the wider population of rural Habiganj district.

### Background characteristics of study participants

Descriptive data on characteristics such as a woman’s religion, her highest level of education, and her height were extracted from the FAARM baseline and endline surveys conducted in 2014 and 2019/2020, respectively. Using the FAARM endline survey's household asset module, the household’s relative position within the 2014 Demographic and Health Survey (DHS) national wealth quintiles was calculated using the Equity Tool [58, 62]. Data on other quantitative descriptive characteristics, such as participant age and current weight, were extracted from the FAARM routine surveillance round at the time of data collection.

### Biosample collection

During the FAARM endline survey, venous blood samples were collected between September and December 2019 from enrolled women (8 mL) and their children aged between 6 months and around 3 years (5 mL), including participants of the MEMAPO and FHEED studies. The primary purpose of blood collection was to assess anemia and micronutrient status [83]. Hematological measurements were performed on-site at the field laboratory. Residual blood samples (serum and EDTA-anticoagulated whole blood) were stored at −20 °C at the field laboratory and subsequently transported to Germany on dry ice (transport time < 24 h) for further micronutrient and additional analyses. Upon arrival, samples were stored at −70 °C at Heidelberg University Hospital.

For the present analysis, FAARM participants—specifically MEMAPO women and children as well as FHEED children—with sufficient remaining blood volume after hematological and micronutrient assessments were included. Only children whose mothers were also enrolled in MEMAPO and had available whole blood mycotoxin data were included in the mother–child pair analyses. In total, samples from 719 participants (433 women and 286 children) were analyzed. Of these, serum samples from 578 participants (421 women and 157 children) were analyzed for aflatoxin-lysine, and whole blood samples from 712 participants (428 women and 257 children) were used for multi-mycotoxin analysis (Table 1). The differing sample sizes reflect variation in the residual sample volumes available for serum (AFB_1_-lysine) and whole blood (multi-mycotoxin) analysis; a small number of participants had sufficient volume for only one matrix (see Table 1 for exact overlap).

**Table 1:** Sociodemographic characteristics of the study participants in rural Habiganj district, Bangladesh, and sample availability

Characteristic	Women (*n* = 433)	Children (*n* = 286)
**Samples available**		
- Whole blood	428	284
- Serum	421	157
**Overlap in sample availability**		
- Both whole blood and serum	416	155
- Only whole blood	12	129
- Only serum	5	2
**Sociodemographic characteristics**		
Age (mean ± SD, range)	26.9 ± 3.9 years (19–38)	12.6 ± 3.6 months (6–21)
Female sex	100%	48%
Weight (mean ± SD, range)	49.3 ± 8.7 kg (29.2–84.4 kg)	8.2 ± 1.2 kg (4.1–13.1 kg)
Religion (%)		
- Muslim	75%	N/A
- Hindu	25%	N/A
Schooling (%)		
- No schooling	14%	N/A
- Only primary (full or partial)	50%	N/A
- Any secondary or higher	36%	N/A
Household wealth quintile^1^		
-Poorest	2%	N/A
-Lower	10%	N/A
-Middle	39%	N/A
-Upper	39%	N/A
-Wealthiest	11%	N/A

^*1*^This is not a relative wealth quintile, but the estimate of the households’ national wealth quintile if the assets owned in 2019 were in the 2014 DHS survey (constructed using www.equitytool.org), *SD* Standard deviation

### Laboratory analyses

#### Chemicals

Methanol and acetonitrile (LC–MS grade) were purchased from Fisher Scientific (Schwerdte, Germany). Formic acid, acetic acid p.a., and Pronase from *Streptomyces griseus* from Merck KGaA (Darmstadt, Germany), ammonia (25%) from VWR (Darmstadt, Germany), and acetone (HPLC grade) were purchased from Carl Roth (Karlsruhe, Germany). Water ASTM type I (> 18 MΩ) was produced with a Purelab Flex system (ELGA, Bielefeld, Germany). Aflatoxin B1 (AFB_1_), aflatoxin B2 (AFB2), aflatoxin G1 (AFG1), aflatoxin G2 (AFG2), aflatoxin M1 (AFM1), BEA, CIT, enniatin A (EnA), enniatin A1 (EnA_1_), EnB, enniatin B1 (EnB_1_), zearalanone (ZAN), and Whatman 903 Protein Saver Cards™ were purchased from Sigma-Aldrich (Taufkirchen, Germany). DH-CIT was obtained from AnalytiCon Discovery GmbH (Potsdam, Germany). Alternariol (AOH), alternariol monomethyl ether (AME), altenuene (ALT), DON, fumonisin B1 (FB1), OTA, ochratoxin α (OTα), 10-hydroxy-ochratoxin A (10-OH-OTA), T-2 toxin (T-2), HT-2 toxin (HT-2), and ZEN were obtained via isolation and purification from fungal cultures [17, 19, 23, 24, 42, 43]. 2’*R*-OTA was produced by thermal degradation as described in Cramer et al. [25]. The glucuronic acid conjugates deoxynivalenol-3-glucuronide (DON-3-GlcA) and HT-2 toxin-4-glucuronide (HT-2–4-GlcA) were enzymatically synthesized using rat and pig liver microsomes [81]. Stable isotope-labeled internal standards d5-OTA, d5-2’*R*-OTA, ^13^C_3_-CIT, and ^13^C_3_-DH-CIT were chemically synthesized according to Cramer et al. [24] and [16].

#### ***Analysis of AFB***_***1***_***-lysine***

AFB_1_-lysine and ^13^C_6_^15^N_2_-AFB_1_-lysine were synthesized according to McCoy et al., and the concentrations were photometrically determined using the molar absorption coefficient of 30,866 M^−1^cm^−1^ for water at pH 7.4 [57, 67, 69].

Sample preparation for aflatoxin-lysine analysis in blood serum was done according to the protocol described by McCoy et al., with slight modifications [57, 65]. 100 to 200 µl of serum, depending on the amount of available material, were mixed with 10 µL formic acid (1%) and 10 µL of the internal standard solution containing 10 ng/mL ^13^C_6_^15^N_2_-AFB_1_-lysine in methanol/water/acetic acid (25/75/0.1, *v/v/v*). 500 µL of Pronase in PBS buffer (6.5 mg/mL, Merck KGaA) was added, and the mixture was incubated for 14 h at 37 °C. 100 µL of PBS was then added, mixed by vortex, and centrifuged at 15,000 × *g* at 8 °C for 5 min. The supernatant was transferred onto a 30 mg OASIS MAX column (Waters, Eschborn, Germany) that had been conditioned with 1 mL methanol and 1 mL water. The supernatant was passed through, followed by 1 mL water, 1 mL methanol (70%), 1 mL ammonium hydroxide in methanol (1%), and 500 µL methanol. Finally, AFB_1_-lysine was eluted by applying 2 × 800 µL of formic acid in methanol (2%). This solution was evaporated to dryness using a rotary vacuum concentrator (Martin Christ Gefriertrocknungsanlagen GmbH, Osterode am Harz, Germany), reconstituted in 200 µL methanol/water/acetic acid (25/75/0.1, *v/v/v*), centrifuged at 15,000 × *g* and 8 °C, 5 min, followed by ultra-high-performance liquid chromatography-tandem mass spectrometry (UHPLC-MS/MS) analysis. With every sample batch, an independent quality control sample of synthetic human serum albumin (HAS)-bound AFB_1_ was analyzed accordingly. This reference was prepared similar to the protocol for AFB_1_-lysine by McCoy et al. [57] but using HSA as a reaction partner instead of lysine. We purified the HSA-bound AFB_1_ by removing unreacted AFB_1_ and low molecular weight reaction products via ultrafiltration using an Amicon Ultra centrifugal filter (Merck) with a cut-off of 10 kDa. The purified reaction product was 10.000-fold diluted with HSA-solution to obtain a concentration range suitable as quality control. Analysis of the HSA-AFB_1_-solution without Pronase treatment was done to confirm the absence of any unbound AFB_1_-lysine. The mean AFB_1_-lysine concentration of the prepared quality control was 1.13 ± 0.094 ng/mL, and the range 0.94—1.27 ng/mL.

Analysis of AFB_1_-lysine samples was carried out on a 1260 Infinity II bio-inert LC system (Agilent, Waldbronn, Germany) coupled to a Sciex 7500 QTRAP activated mass spectrometer (Sciex, Darmstadt, Germany) [65]. For chromatographic separation, a Reprosil Pur C18AQ column (2.0 × 150 mm, 3 µm) equipped with a guard column of the same material (2.0 × 5 mm) (both Dr. Maisch GmbH, Ammerbuch, Germany) was used. The following linear gradient of water (A) and acetonitrile (B), both containing 0.01% acetic acid, was used at a flow rate of 450 µL/min: 0 min 5% B, 3 min 5% B, 10 min 70% B, 10.1 min 95% B, 12.0 min 95% B, 12.1 min 5% B, 15 min 5% B. Column temperature was held at 45 °C. The injection volume of the UHPLC system was 40 µL.

The analytes were ionized by positive electrospray at 2500 V, with the source temperature (Gas 2) set to 550 °C. Curtain gas, nebulizer gas (Gas 1), and drying gas (Gas 2) were set to 45 psi, 65 psi, and 75 psi, respectively. The collision gas for fragmentation was set to an instrument value of 8 arbitrary units. Measurements were performed in a combination of MRM and MS^3^ experiments with a total cycle time of 350 ms. MS^3^-Experiments were applied in order to obtain a sufficiently sensitive and selective confirmatory mass transition in the low concentration range as the other MRM transitions listed below showed much lower signal intensities. For AFB_1_-lysine, the mass transitions m/z 457.2/394.1, and for the internal standard, m/z 465.2/400.1, (both at a collision energy voltage of 27 V) were used for quantification, with a dwell time of 20 ms each. Qualification was done by monitoring the consecutive mass transition m/z 457.2/394.1/348.1 in the ion trap of the mass spectrometer with the following parameters being applied: Collision energy voltage: 27 V, Auxiliary voltage: 0.26 V, fixed fill time: 80 ms, scan range (MS^3^): m/z 200–380, scan rate 10,000 Da/s. The following mass transitions were additionally recorded with collision energy voltages given in brackets: 457.2/376.1 (45 V), 457.2/328.0 (42 V), 457.2/411.0 (15 V), 465.2/382.1 (45 V), 465.2/329.1 (42 V), 465.2/419.0 (15 V). Data acquisition and analysis were performed using Sciex OS 3.1 software.

#### Calibration

Calibration was prepared in acetonitrile/water/acetic acid (5/95/0.01, *v/v/v*) as an eight-point calibration over the concentration range 5 pg/mL to 5 ng/mL, with an internal standard concentration of 0.1 ng/mL. The limit of detection (LOD) was 15 pg/mL and the limit of quantification (LOQ) was 40 pg/mL, determined by signal-to-noise ratios of 3 and 10, respectively, in matrix samples. Average recovery for 0.1 ng/mL and 1 ng/mL was 116.3% ± 9.3%.

#### Analysis of multiple mycotoxins in dried blood spots

Analysis of mycotoxins and mycotoxin metabolites was performed as described previously in [63] and [26]. Briefly, 100 µL of whole blood were spotted onto Whatman 903 Protein Saver cards™ and dried overnight at room temperature. After cutting out the spot, 1 mL of water/acetone/acetonitrile (30/35/35, *v/v/v*) and 10 µL of an internal standard solution containing d5-OTA, d5-2’R-OTA, 13C3-CIT, and 13C3-DH-CIT were added, and the mixture was ultrasonicated for 30 min. An aliquot of 700 µL was evaporated to dryness under vacuum. The residue was reconstituted in 100 µL of water/acetonitrile/formic acid (95/5/0.1, v/v/v), centrifuged at 15,000 × g for 5 min, and the supernatant was analyzed by UHPLC-MS/MS. A quality control sample of fortified whole blood was analyzed with each sample batch. Mean concentrations of OTA, 2’*R*-OTA, CIT, and DH-CIT were 0.11 ± 0.02, 0.023 ± 0.004, 0.22 ± 0.05, and 0.16 ± 0.01 ng/mL with ranges of 0.09—0.16, 0.016—0.031, 0.12—0.31, and 0.13—0.19 ng/mL, respectively.

Samples were analyzed using a 1260 Infinity II Bio-inert LC system (Agilent) coupled to a Sciex 7500 QTRAP activated mass spectrometer (Sciex). Analyte separation was done on a Nucleodur C_18_ Gravity-SB column (2.0 × 100 mm, 3 µm) equipped with a guard column of the same material (2.0 × 4 mm) (both Macherey–Nagel). The following linear gradient of water (A) and acetonitrile (B), both containing 0.1% formic acid, was used at a flow rate of 500 µL/min: 0 min 5% B, 1 min 5% B, 12 min 95% B, 14.5 min 95% B, 14.51 min 5% B, 17 min 5% B. Column temperature was held at 45 °C. The injection volume of the UHPLC system was 30 µL.

The analytes were ionized by positive and negative electrospray ionization in a single run. The ion voltage was held at ± 3000 V, and the source temperature (Gas 2) was set to 600 °C. Curtain gas, nebulizer gas (Gas 1), and drying gas (Gas 2) were set to 45 psi, 60 psi, and 60 psi, respectively. The gas for collision fragmentation was set to an instrument value of 9 arbitrary units. Measurements were performed in scheduled multiple reaction monitoring (sMRM) with a target cycle time of 200 ms and a retention window of 15–40 s to obtain maximum sensitivity due to optimized dwell times For each compound, one sMRM transition was used for quantification, while one or two additional transitions were monitored for qualification. More detailed information on the MRM parameters is given in Table S1 (Supplementary Material). Data acquisition and analysis were done using Sciex OS 3.1 software.

Solvent calibration with internal standards was prepared in water/acetonitrile/formic acid (95/5/0.1/, *v/v/v*) as a ten-point 1/x weighted calibration. Quantification was performed for OTA, 2’R-OTA, CIT, and DH-CIT, with respective limits of detection of 0.003, 0.003, 0.007, and 0.003 ng/mL and limits of quantification of 0.01, 0.01, 0.02, and 0.01 ng/mL. The remaining analytes were monitored qualitatively with respective limits of detection given in brackets: AFB_1_ (0.006 ng/mL), AFB_2_ (0,013 ng/mL), AFG_1_ (0.014 ng/mL), AFG_2_ (0.027 ng/mL)AFM_1_ (0.014 ng/mL), ALT (0.08 ng/mL), AME (0.146 ng/mL), AOH (0.142 ng/mL), Bea (0.013 ng/mL), DON (0.3 ng/mL), DON-3-GlcA (1.3 ng/mL), EnA (0.002 ng/mL), EnA_1_ (0.003 ng/mL) EnB (0.001 ng/mL), EnB_1_ (0.004 ng/mL) FB_1_ (0.6 ng/mL) OTα (0.01 ng/mL), 10-OH-OTA (0.01 ng/mL), HT-2 (1.4 ng/mL), HT-2–4-GlcA (0.7 ng/mL), T-2 (0.2 ng/mL), ZAN (0.3 ng/mL) and ZEN (0.3 ng/mL). Limits of detection and quantification were determined by signal-to-noise ratios of 3 and 10 in matrix samples. Validation of the method for OTA and 2’R-OTA was previously published and reconfirmed during measurement. For CIT and DHCIT, where initial methods were validated without internal standards, a mean recovery of 74.9 ± 2.49% and 98.1 ± 3.35% was determined, respectively.

### Statistical analysis

#### Management of left censoring and descriptive analysis

Left-censored blood mycotoxin concentrations, i.e., concentrations below the LOD and LOQ of the analytical method, were handled using the substitution methods in accordance with the European Food Safety Authority (EFSA) guidance [31]. In line with EFSA recommendations for substances with high occurrence, only the middle-bound (MB) approach was applied, as OTA was detected in nearly all samples and CIT in 91% of samples, resulting in a very low proportion of left-censored observations. Under the MB scenario, concentrations below the LOD were substituted with LOD/2, while concentrations below the LOQ were substituted with LOQ/2. Descriptive statistics were subsequently calculated using the imputed MB dataset. For AFB_1_-lysine, descriptive analyses were based on observed detection frequencies and quantifiable concentrations only, and no substitution approach was used for main descriptive statistics due to the high proportion of left-censored observations, particularly among children. For the exploratory correlation analysis with whole blood OTA in the subsample, a lower-bound approach was applied, where non-detects were assigned zero, and interval-censored values (> LOD and < LOQ) were assigned the LOD.

#### Estimation of dietary mycotoxin exposure

The toxicokinetic models used here back-calculate an estimated daily intake from measured blood concentrations. Dietary intake is the dominant route of exposure to OTA and CIT according to the literature, and because non-dietary contributions to blood mycotoxin levels are not well quantified for this population, we refer to the resulting estimates as 'probable daily intake (PDI)' following established usage in the toxicokinetic literature [80], while acknowledging that this approach implicitly attributes the entirety of measured systemic exposure to dietary sources. To the extent that inhalation or dermal exposure contribute to measured blood concentrations, our PDI estimates would overestimate true dietary intake, though total exposure estimates remain valid. Dietary exposure was assessed by estimating the PDI of OTA and CIT, the two most frequently detected mycotoxins in the study population. The methodology applied for these two mycotoxins is described below.

#### OTA intake estimation

The PDI of OTA was calculated using two toxicokinetic models proposed in the literature [80], both derived from the Klaassen equation describing the relationship between total body clearance and steady-state plasma concentration [50]. Model 1, the most commonly applied approach for intake estimation based on serum OTA concentrations, has been widely used in previous studies [10, 22, 30, 72] and is based on a human toxicokinetic study by Studer-Rohr, who compared renal and plasma clearance of OTA and concluded that non-renal elimination pathways were negligible [73]. Under standard assumptions (70 kg body weight, plasma clearance of 0.048 mL/min, bioavailability of 0.5), this yields:

1 $${k}_{0}=1.97\times \mathrm{C}\mathrm{p}$$

where k_0_ is the daily intake of OTA (ng OTA/kg body weight/day), and Cp is the plasma concentration of OTA (ng/ml). The same human kinetic study was subsequently republished with a recalculated, higher renal clearance estimate of 0.1099 mL/min [74], giving an alternative coefficient:

2 $${\mathrm{k}}_{0}=4.52\times \mathrm{C}\mathrm{p}$$

Given that most published studies rely on Model 1, but updated toxicokinetic data suggest a higher clearance value, both models were applied in the present analysis as previously done by [80], to allow comparison and to account for uncertainty in OTA toxicokinetic parameters. Full derivations of both coefficients are provided in Supplementary Methods S1 (section S1.1). In our study, individual body weights of participants were available and were therefore incorporated directly into the original equation rather than the standard body weight of 70 kg commonly used in the literature; this was considered important given that the average body weight in our study population was substantially lower, at about 49 kg for women and about 8 kg for children.

#### CIT intake estimation

CIT intake was estimated using a toxicokinetic approach based on the Klaassen equation, applying parameters by  Degen et al.  [28]:

$${\mathrm{k}}_{0}=\frac{Clp \times Cp}{A} \times\uptau$$

where k_0_ is the daily intake, *Clp* is the plasma clearance, *Cp* is the plasma concentration, A is the oral bioavailability (fraction absorbed), and τ (tau) is the dosing interval. Plasma clearance (Clp) was derived from an oral dosing study as Clp = (D/AUC) × f, where D is the administered dose, AUC is the area under the plasma concentration–time curve, and f is the fraction absorbed, estimated from the cumulative urinary excretion of citrinin and its metabolite. Substituting this expression into the Klaassen equation and cancelling the bioavailability term (f) yields a simplified intake equation that does not require independent estimation of f (full derivation in Supplementary Methods S1). Using a dosing interval of 1440 min (24 h) and normalizing by body weight, the final intake equation is:

$${\mathrm{k}}_{0}=11.5/bodyweight\times Cp\times 1440$$

#### Health risk characterization

Two complementary approaches were applied to assess the health risks associated with the two frequently occurring mycotoxins, OTA and CIT, which differ in their toxicological profiles. OTA is classified by IARC as a Group 2B carcinogen (possibly carcinogenic to humans) [44, 45] although its carcinogenic mechanism remains incompletely characterized, EFSA's risk assessment notes that the evidence for direct genotoxic interaction with DNA is inconclusive [36]. Nonetheless, EFSA has applied the Margin of Exposure approach typically reserved for genotoxic carcinogens as a precautionary measure, given that a non-genotoxic mechanism cannot be excluded [33, 34]; we follow this precautionary framework here, while noting that it may represent a conservative approach that could overestimate risk relative to a threshold-based (non-MOE) assessment should OTA's carcinogenicity not involve direct genotoxicity.

The MOE was calculated as the ratio of the benchmark dose lower confidence limit for a 10% response (BMDL_10_​) to individual exposure estimates, expressed as PDI. According to the Scientific Committee of EFSA and the World Health Organization, an MOE value of 10,000 or higher is considered of low concern to public health [34]. It is noteworthy, however, that the MOE approach is intended as a screening and prioritization tool to identify exposures warranting risk management attention, rather than a direct quantitative estimate of risk. Hence, an MOE below 10,000 indicates that further evaluation or risk management measures may be warranted, not that harm has occurred or will occur with a certain quantifiable probability [34]. For OTA, a BMDL_10_ of 14.5 µg/kg body weight/day, based on neoplastic effects, was used in the MOE calculations [36]. For CIT, which is a non-genotoxic mycotoxin with an established health-based guidance value, risk was assessed using the hazard quotient (HQ) approach. HQ was calculated by comparing individual PDIs with the tolerable daily intake (TDI). The TDI applied was 200 ng/kg body weight/day for CIT as established by EFSA [32]. When the HQ was less than 1, the exposure was considered within safe limits.

## Results

### Description of the study population

Characteristics of the study population in rural Habiganj, Bangladesh, are summarised in Table 1. Briefly, the analytical sample comprised 433 women and 286 children, with availability of whole blood samples in both groups and more limited availability of serum among children. Women were young adults, with a mean age of about 27 years at the time of sampling, while children were predominantly in late infancy (mean age 13 months). Three quarters of women were Muslim and had at least partial primary education (86%) and only 36% had any secondary education or higher. Household wealth distribution, based on endline measurements in 2019/2020, was concentrated in the middle and upper quintiles of the 2014 Bangladesh Demographic and Health Survey wealth index [62], with relatively few participants in the poorest category. Among children, sex distribution was balanced (52% male, 48% female).

### Occurrence of mycotoxin biomarkers in blood

Tables 2 and 3 summarise the occurrence and concentrations of mycotoxin biomarkers and metabolites in whole blood and serum samples from women and children. Overall, exposure to multiple mycotoxins was widespread. In whole blood, OTA was detected above the LOQ in 100% of samples from both women and children, with high co-occurrence (> 97%) of its diastereomer 2′*R*-ochratoxin A (2′*R-*OTA). CIT was also highly prevalent (> 90% detected), whereas its metabolite DH-CIT was detected less frequently (24% overall). Several emerging mycotoxins, particularly EnB, were frequently detected (92%); data for EnB and BEA, included as analytes amenable to blood-based detection, are provided in Supplementary Table S4.

**Table 2:** Occurrence and concentration of quantified mycotoxin biomarkers in human whole blood samples from rural Habiganj district, Bangladesh

**Mycotoxin biomarker**	**Instrumental limits for biomarkers (ng/ml)**		**Analyzed samples**	**Detected, n (% ≥ LOD)**	**Quantified, n (% ≥ LOQ)**	**Mean (ng/mL)**	**Range (ng/mL)**
	LOD	LOQ					
CIT	0.007	0.02					
women			428	390 (91.1)	252 (58.9)	0.398	0.022–4.918
children			284	257 (90.5)	126 (44.4)	0.196	0.021–3.232
All			712	647 (90.9)	378 (53.1)	0.331	0.021–4.918
DH-CIT	0.003	0.01					
women			428	126 (29.4)	(11.2)	0.057	0.012–0.286
children			284	44 (15.5)	(4.6)	0.079	0.013–0.363
All			712	170 (23.9)	(8.6)	0.062	0.012–0.363
OTA	0.003	0.01					
women			428	428 (100)	428 (100)	0.542	0.079–3.541
children			284	284 (100)	284 (100)	0.303	0.034–7.932
All			712	712 (100)	712 (100)	0.446	0.034–7.932
2’R-OTA	0.003	0.01					
women			428	413 (96.5)	299 (69.9)	0.103	0.011–1.429
children			284	281 (98.9)	244 (85.9)	0.158	0.117–4.726
All			712	694 (97.5)	543 (76.3)	0.128	0.011–4.726

*LOD* Limit of detection, *LOQ* Limit of quantification, *OTA* Ochratoxin A, *2’R-OTA* 2'R-Ochratoxin A; *CIT* Citrinin, *DH-CIT* Dihydrocitrinone

**Table 3:** Occurrence and concentration of aflatoxin B1 biomarker in serum samples from rural Habiganj district, Bangladesh

**Mycotoxin biomarker**	**Instrumental limits for biomarkers (ng/mL)**		**Analyzed samples**	**Detected, n (% ≥ LOD)**	**Quantified, n (% ≥ LOQ)**	**Mean (ng/mL)**	**Range (ng/mL)**
	LOD	LOQ					
AFB_1_-lysine	0.015	0.04					
women			421	95 (22.6)	14 (3.3)	0.050	0.030–0.096
children			157	6 (3.8)	1 (0.6)	0.032	-
All			578	101(17.5)	15 (2.6)	0.049	0.030–0.096

*LOD* Limit of detection, *LOQ* Limit of quantification, *AFB*_*1*_*-lysine* Aflatoxin B_1_-lysine adduct

Whole blood concentrations of CIT and its primary metabolite DH-CIT were moderately correlated (Spearman’s *ρ* = 0.53; *n* = 712; *p* < 0.001), indicating a consistent co-occurrence of the parent compound and metabolite in circulation across the study population. In contrast, though OTA was detected in nearly all whole blood samples, its concentrations showed only a weak correlation with CIT concentrations (Spearman’s *ρ* = 0.28; *n* = 712; *p* < 0.001), indicating partially overlapping but largely independent exposure patterns between these mycotoxins (Table S2). However, the longer biological half-life of OTA compared to CIT has to be considered. To assess whether OTA and CIT exposure reflect shared household dietary sources, we examined whole blood concentrations within mother–child pairs (*n* = 87 dyads, identified from the 284 children with available mycotoxin data, linked to their respective mothers). Whole blood OTA concentrations were moderately correlated within mother–child pairs (Spearman's *ρ* = 0.47, *n* = 85, *p* < 0.001), consistent with sustained, shared dietary exposure within the household. In contrast, whole blood CIT concentrations showed no within-dyad correlation (Spearman's *ρ* = 0.01, *n* = 68, *p* = 0.913) (Table S3).

Serum analysis found that 22.6% of women and 3.8% of children had detectable levels of the AFB_1_-albumin adduct AFB_1_-lysine (Table 3), demonstrating ongoing AF exposure. Quantifiable AFB_1_-lysine concentrations were observed in a small proportion of samples (3.3% of women and 0.6% of children), with mean concentrations of 0.05 ng/mL among women. Using a harmonised analytic sample comprising women and children with both serum and whole blood measurements, and applying a lower-bound substitution approach for serum AFB_1_-lysine—where non-detects were set to zero, and interval-censored values (> LOD and < LOQ) were assigned the LOD—whole blood OTA and AFB_1_-lysine concentrations showed a weak positive correlation (Spearman’s *ρ* = 0.24; *n* = 571; *p *< 0.001) (Table S2).

Figure S1 illustrates the high prevalence of co-exposure to multiple blood-detectable mycotoxins in the study population. The most common profile was concurrent detection of total ochratoxin A (tOTA; representing the sum of OTA and its isomer 2’R-OTA), total citrinin (tCIT; comprising CIT and its degradation product DH-CIT), and EnB, observed in 79% of participants. Less frequent patterns included co-occurrence of tOTA and EnB only (7%) and tOTA with tCIT (7%). Additional exposure profiles comprised simultaneous detection of tOTA, tCIT, EnB, and BEA (5%). Rare patterns included tOTA alone (1%), tOTA with EnB and BEA (0.6%), and tOTA with tCIT and BEA (0.1%).

**Fig. 1:**
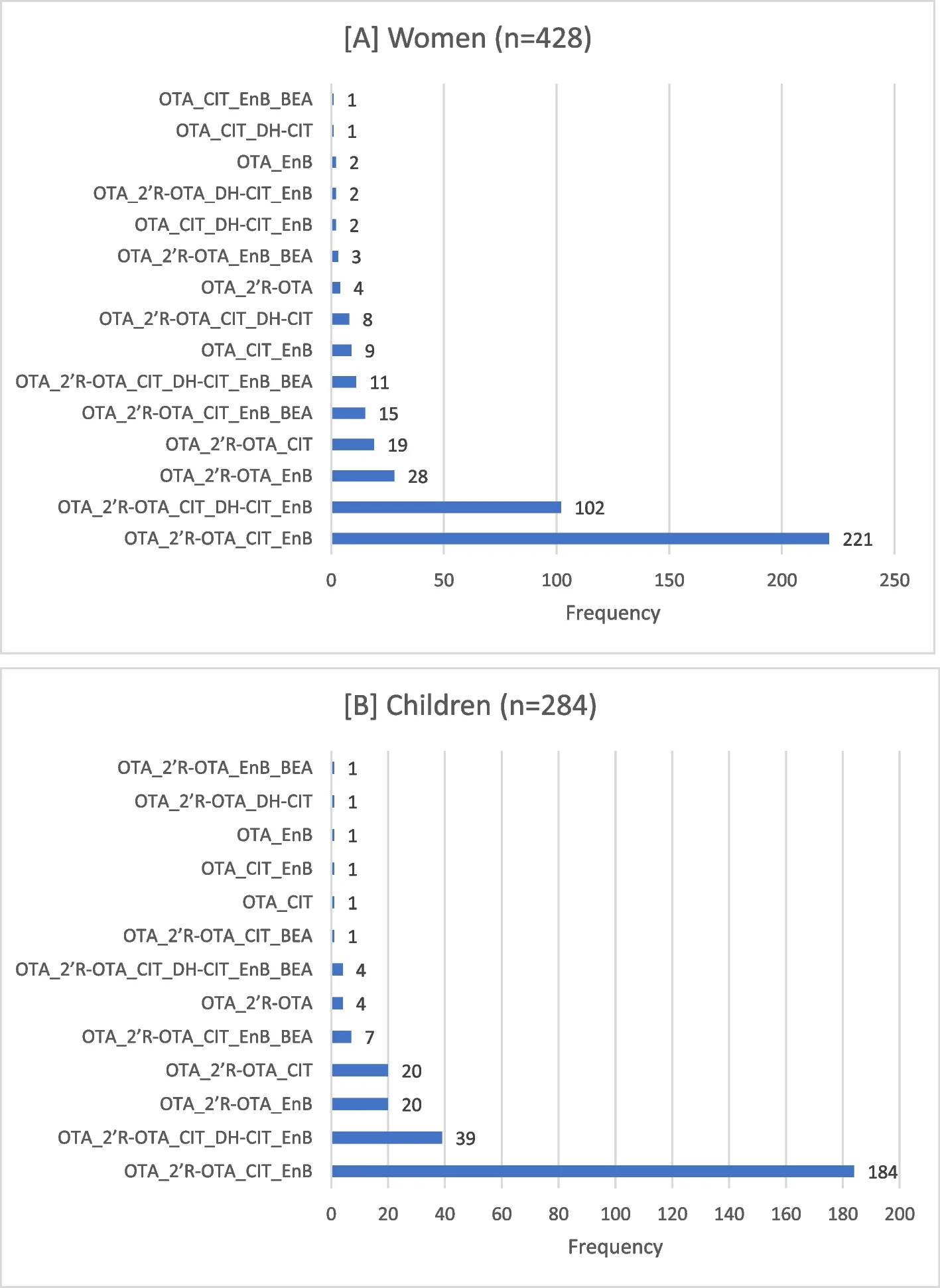
Mycotoxin and metabolite co-occurrence in whole blood samples of women (**A**) and children (**B**) in rural Bangladesh. The most prevalent co-exposure pattern in both groups was the combination of ochratoxin A (OTA), 2'R-ochratoxin A (2’R-OTA), citrinin (CIT), and enniatin B (ENB) — found in 221 women (52%) and 184 children (65%). Abbreviations: OTA: Ochratoxin A; 2’R-OTA: 2'R-Ochratoxin A; CIT: Citrinin; DH-CIT: Dihydrocitrinone; EnB: Enniatin B; BEA: Beauvericin

Overall, women tended to have a wider range of detected biomarkers and metabolites per sample than children, suggesting potential differences in dietary exposure profiles or intake diversity. The distribution of different combinations of mycotoxins and metabolites in the whole blood of women and children is illustrated in Fig. 1. Across both groups, the most frequent combinations involved OTA, 2′R-OTA, and CIT, often co-occurring with additional mycotoxins such as EnB, DH-CIT, and BEA. Taken together, these findings indicate widespread and complex multi-mycotoxin exposure in the study population, predominantly driven by OTA-related co-exposure mixtures.

### Dietary mycotoxin exposure and health risk assessment

**Fig. 2:**
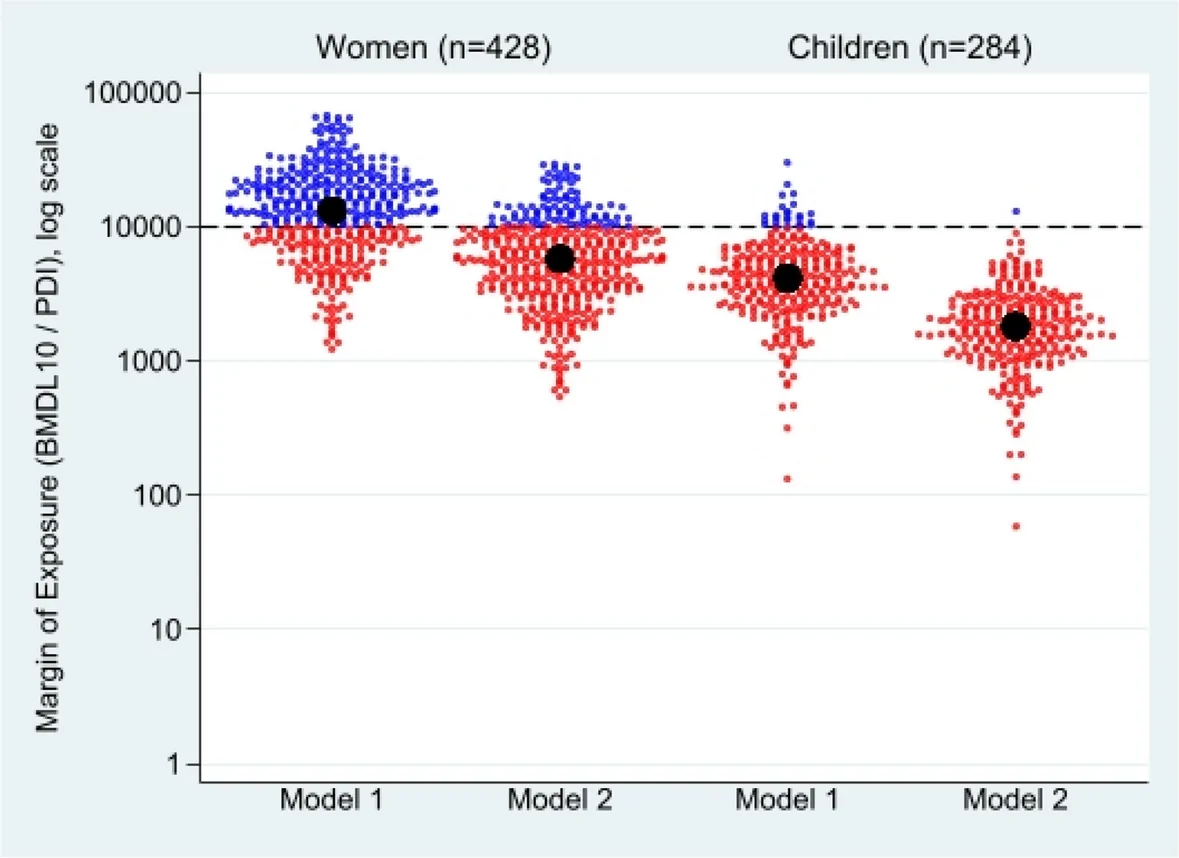
Margin of Exposure to dietary ochratoxin A among women and children in rural Bangladesh, by toxicokinetic model. The Margin of Exposure (MOE) is calculated by dividing the benchmark dose lower confidence limit (BMDL_10_) for ochratoxin A (OTA; 14.5 µg/kg body weight/day) by each participant's probable daily intake (PDI), shown on a logarithmic y-axis. Each point represents one participant; black circles indicate group medians. Horizontal position within each group reflects local point density (beeswarm arrangement) and does not represent any measured variable. PDI was back-calculated from measured plasma OTA concentrations using the Klaassen equation (k0 = Clp × Cp/A, where Clp is plasma clearance, Cp is plasma concentration, and A is the bioavailability of OTA), applying two plasma clearance estimates: 0.048 mL/min (Model 1) and 0.1099 mL/min (Model 2). Because estimated intake is directly proportional to clearance, the higher clearance value in Model 2 yields higher back-calculated PDI and particularly in a warm and humid climaingly lower (more conservative) MOE estimates. Blue points indicate MOE ≥ 10,000 (low health concern); red points indicate MOE < 10,000 (potential concern). Unlike the hazard quotient in Fig. 3, higher MOE values indicate lower health concern. The dashed horizontal line indicates the EFSA/WHO threshold of low health concern (MOE = 10,000). In our sample, 37% (Model 1) and 80% (Model 2) of women, and 91% (Model 1) and 99% (Model 2) of children had MOEs below this threshold

**Fig. 3:**
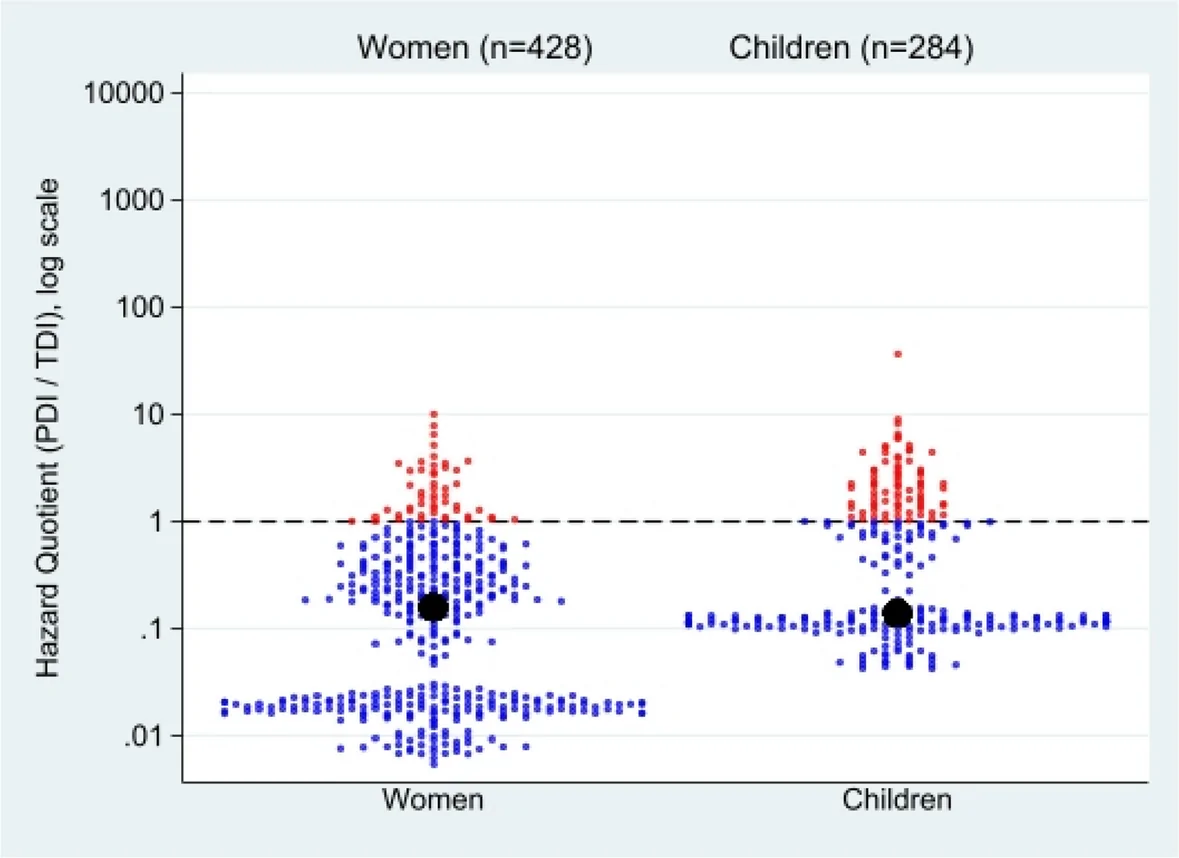
Hazard quotient (HQ) of dietary citrinin exposure among women and children in rural Bangladesh. The Hazard quotient (HQ) is calculated by dividing the individual probable daily intake (PDI) by the established tolerable daily intake (TDI) for citrinin (200 ng/kg body weight/day). Each point represents one participant, shown on a logarithmic y-axis; dark circles indicate group medians. Horizontal position within each group reflects local point density (beeswarm arrangement) and does not represent any measured variable. Blue points indicate HQ ≤ 1 (within tolerable limits); red points indicate HQ > 1 (potential concern). The dashed horizontal line marks HQ = 1; values above this line may indicate a potential health concern requiring prioritization for further attention. In our sample, 12% of women and 27% of children exceeded this threshold. Horizontal banding at low HQ values reflects a shared substituted value assigned to citrinin measurements below the analytical limit of detection, rather than true biological variation

The estimated daily intake of OTA and CIT, along with their health risk characterization, is presented in Table 4. Among women (*n* = 428), mean PDI values for OTA were 1.6 ng/kg bw/day (Model 1) and 3.7 ng/kg bw/day (Model 2), with 37% and 80% exceeding the health concern thresholds, respectively (Fig. 2). For CIT, the mean PDI was 88.7 ng/kg bw/day, with 12% of women exceeding the tolerable daily intake (Fig. 3). Among children (n = 284), mean PDI values for OTA were higher, at 5.1 ng/kg bw/day (Model 1) and 11.6 ng/kg bw/day (Model 2), with the majority exceeding the health concern thresholds —91% and 99%, respectively (Fig. 2). For CIT, the mean PDI was 201.1 ng/kg bw/day, and 27% of children exceeded the tolerable daily intake (Fig. 3).

**Table 4:** Estimated probable daily intake and risk characterization of ochratoxin A and citrinin among women and children in rural Bangladesh

Sample	Mycotoxin	Probable Daily Intake (ng/kg bw/day)		Health concern* n (%)
		**Mean ± SD**	**Range**	
Women (n = 428)	OTA (Model 1)	1.6 ± 1.6	0.2–11.8	157 (37)
	OTA (Model 2)	3.7 ± 3.7	0.5–27.0	342 (80)
	CIT	88.7 ± 188.9	1.1–1997.6	51 (12)
Children (n = 284)	OTA (Model 1)	5.1 ± 7.8	0.5–109.1	259 (91)
	OTA (Model 2)	11.6 ± 17.8	1.1–249.8	283 (99)
	CIT	201.1 ± 500	8.4–7244.8	78 (27)
Women and children (n = 712)	OTA (Model 1)	3.0 ± 5.3	0.2–109.1	416 (58)
	OTA (Model 2)	6.8 ± 12.2	0.5–249.8	625 (88)
	CIT	133.6 ± 352.1	1.1–7244.8	129 (18)

Middle-bound substitution was applied to CIT alone; no substitution was necessary for OTA; Model 1 result (using a clearance estimate of 0.048 ml/min); Model 2 result (using a clearance estimate of 0.1099 ml/min); *Health concern: Hazard quotient > 1 for CIT and Margin of Exposure < 10,000 for OTA; Tolerable daily intake – for CIT: 200 ng/kg bw; *OTA* ochratoxin A, *CIT* citrinin, *bw* body weight, *SD* Standard deviation

In the total population of women and children (n = 712), mean OTA PDIs were 3.0 ng/kg bw/day (Model 1) and 6.8 ng/kg bw/day (Model 2), with 58% and 88% of participants exceeding the health concern thresholds, respectively (Fig. 2). Mean CIT PDI was 133.6 ng/kg bw/day, with 18% of the population exceeding the TDI (Fig. 3). Overall, children were more highly exposed than women, and OTA exposure posed a greater health concern than CIT, particularly when estimated using Model 2.

## Discussion

In this study, we provide blood-based mycotoxin biomarker results from a cohort of women and young children in rural Bangladesh with previously reported urinary mycotoxin exposure [52]. Our results demonstrate widespread and chronic co-exposure to multiple mycotoxins, dominated by OTA and CIT-related compounds, alongside frequent detection of emerging mycotoxins such as enniatin B. Importantly, not only OTA, but also its diastereomer 2′R-OTA was detected in almost all women and children. We also detected the aflatoxin B_1_ adduct AFB_1_-lysine in serum which confirms ongoing, albeit generally low-level AFB_1_ exposure. Our dietary exposure assessment based on blood biomarkers indicates that OTA warrants further evaluation or risk management measures in our study population, particularly among children, with over 90% of children exceeding the health concern threshold.

OTA was detected and quantifiable in 100% of whole blood samples from both women and children, with a similarly high occurrence of its diastereomer 2′*R*-OTA. This universal detection provides compelling evidence of chronic, sustained OTA exposure, extending and strengthening earlier findings from the same cohort based on urinary biomarkers [52]. In the previous study that examined urine samples of women during early pregnancy, taken between July 2018 and November 2019, OTA was the most prevalent mycotoxin and was identified as the primary contributor to dietary risk. The present results, based on blood samples of women and young children taken from September to December 2019, confirm that this pattern is not driven by short-term dietary variability. Given OTA's long half-life (~ 35 days) and extensive plasma protein binding, these blood-based results reflect a cumulative systemic burden, indicating that exposure is persistent rather than transient.

An important and novel observation of the present study is the unexpected prominence of 2′R-OTA, which was detected in over 97% of individuals and frequently at quantifiable concentrations. 2′R-OTA is a heat-induced reaction product of OTA formed through stereochemical conversion of the phenylalanine moiety from the L- to the D-configuration, a process known to occur during intense thermal treatment, such as roasting or prolonged heating at temperatures ≥ 100 °C [25, 76]. In European biomonitoring studies, 2′R-OTA has been strongly associated with coffee consumption and has not been detected in non-coffee drinkers or in children, supporting coffee as the primary dietary source in European populations [25, 75, 80].

In contrast, coffee consumption is relatively uncommon in rural Bangladesh, particularly among women and young children, and is thus unlikely to explain the high prevalence and concentrations of 2′R-OTA observed in this cohort. The widespread detection of 2′R-OTA in both mothers and children therefore suggests the existence of alternative dietary sources or exposure pathways, potentially related to local food processing and cooking practices. Traditional Bangladeshi diets commonly involve prolonged boiling, roasting, frying, or reheating of staple foods, spices, and dried or fermented products, which may facilitate the thermal transformation of OTA into 2’R-OTA when contaminated raw ingredients are subjected to extended heat treatment. The near-universal co-occurrence of OTA and 2’R-OTA observed in this study is consistent with this hypothesis and raises the possibility that household-level food preparation may substantially modify the mycotoxin exposure profile. We emphasize that this explanation is hypothesis-generating rather than confirmatory: the present study did not collect food consumption or food contamination data, and we therefore cannot directly link 2′R-OTA levels to specific cooking practices or food items at the individual level. Alternative explanations should also be considered, including the possibility that 2′R-OTA forms during industrial or small-scale commercial food processing (e.g., parboiling or drying of rice or other staples) prior to household acquisition, rather than during household cooking itself, or that it derives from a food source not yet identified as contributing to OTA exposure in this setting. Targeted studies that pair food contamination and consumption data with biomarker analyses are needed to test this hypothesis.

From a toxicological perspective, the prominence of 2′R-OTA is noteworthy. Although in vitro data suggest that 2′R-OTA is approximately ten times less cytotoxic than OTA, it remains toxic to human kidney epithelial cells at nanomolar concentrations; however, its in vivo relevance and contribution to nephrotoxicity remain incompletely characterized [25]. Given the chronic nature of exposure observed in this population and the frequent co-occurrence with OTA and CIT, the health implications of long-term exposure to 2′R-OTA—particularly during sensitive developmental periods—remain insufficiently understood. Current risk assessments and regulatory frameworks do not account for this diastereomer, potentially leading to underestimation of ochratoxin-related health risks in populations where heat-processed foods constitute a major component of the diet.

CIT was also highly prevalent in whole blood (> 90%), whereas its metabolite dihydrocitrinone (DH-CIT) was detected less frequently. This pattern likely reflects differences in toxicokinetics and metabolic conversion, with parent compounds persisting longer in circulation than their metabolites. Consistent with this, whole blood concentrations of CIT and DH-CIT were moderately correlated, supporting a shared dietary exposure pathway while highlighting substantial inter-individual variability in metabolism and clearance. The incomplete correlation indicates that DH-CIT provides complementary rather than redundant information to CIT, capturing aspects of metabolic processing rather than overall exposure magnitude. We note that, given the large sample size in this study (n = 571 to 712), even modest correlation coefficients reach high statistical significance; the correlations between OTA and CIT (ρ = 0.28) and between AFB_1_-lysine and OTA (ρ = 0.24) should therefore be interpreted as indicating partially overlapping but largely independent exposure patterns, rather than a strong shared dietary source (Supplementary Figure S2). The weak correlation between whole blood OTA and CIT likely stems from their different toxicokinetics: OTA's long biological half-life reflects exposure over the last month, while CIT blood levels are mostly linked to the last one or two meals. This distinction is further supported by our mother–child dyad-level analysis: whole blood OTA concentrations between mothers and their own children were moderately correlated (ρ = 0.47), consistent with OTA's long half-life capturing a shared, cumulative dietary exposure over the preceding weeks. By contrast, maternal and child CIT concentrations showed no association (ρ = 0.014), in keeping with CIT's much shorter half-life, such that a single blood sample reflects recent, individually variable intake (e.g., differing meal timing or portion-specific contamination) rather than a stable shared exposure level.

While a strong correlation would point to a shared food source, the modest association between OTA and CIT suggests that co-detection reflects independent, overlapping dietary exposures rather than a single contaminated item. Furthermore, the frequent co-occurrence of OTA, 2′R-OTA, and CIT highlights sustained exposure to multiple nephrotoxic mycotoxins, raising concerns about potential additive or synergistic effects.

Given that both AFB_1_-lysine and OTA serve as longer-term exposure biomarkers, their modest correlation suggests that co-occurrence reflects partially overlapping dietary exposure, possibly from rice or spices, rather than intake from a single, consistently co-contaminated source, with their distinct toxicokinetic profiles likely also driving the observed mismatch in blood concentrations. Experimental evidence suggests that combined exposure to OTA and CIT may exacerbate renal toxicity, emphasizing the importance of considering mixture effects when looking at health outcomes rather than evaluating individual mycotoxins in isolation [38]. The present risk characterization was conducted separately for OTA and CIT, in line with most published mycotoxin risk assessments, but this likely underestimate real-world risk given the near-universal co-exposure observed here. Cumulative risk assessment approaches — such as the hazard index (summing individual hazard quotients for compounds sharing a common toxicological endpoint, e.g., nephrotoxicity) or relative potency factor methods [55] — could in principle be applied to OTA and CIT given their shared nephrotoxic mechanism, but require dose–response data for combined exposure that are not yet well established for this combination. We were therefore unable to apply such a framework with confidence in the present analysis. We highlight this as a priority for future work, particularly given experimental evidence of additive or synergistic nephrotoxic effects from combined OTA and CIT exposure [38].

A key finding of this study is the near-universal exposure to multiple mycotoxins among both women and children. None of the whole blood samples was free from the investigated mycotoxins. The most common exposure pattern involved OTA, 2′R-OTA, and CIT, often in combination with DH-CIT, enniatin B, or beauvericin. These findings closely mirror, but also extend, the earlier urine-based study, which identified OTA–CIT co-exposure as the dominant pattern during early pregnancy [52]. Importantly, the high prevalence in blood implies that such co-exposures are not transient, but persist over longer time windows, reinforcing concerns about combined toxicity. Given evidence from experimental studies that OTA and CIT may exert additive or synergistic nephrotoxic effects, the observed co-occurrence patterns raise important questions about health risks not captured by single-compound risk assessments.

We also examined so-called emerging mycotoxins [40], for which no regulations exist as they have only recently been recognized as a risk, and several, particularly enniatin B, were frequently detected in the whole blood samples of our study population. The high prevalence of enniatin B in both women and children suggests regular dietary exposure, consistent with its increasing detection in cereals and cereal-based foods globally. While toxicological data on enniatins remain limited, their widespread presence alongside regulated mycotoxins [40] underscores the importance of broad-spectrum biomonitoring approaches and the need for further research on potential health effects, especially under conditions of chronic co-exposure.

The present analysis highlights the value of measuring the AFB_1_-lysine adduct, a well-established biomarker of longer-term aflatoxin exposure. While AFM_1_ was detected in only a few whole blood samples (≈2%), this short-term biomarker is primarily excreted in urine and is not reliably captured in blood. In contrast, AFB_1_-lysine was detected in nearly one-quarter of women and a smaller proportion of children, clearly indicating ongoing exposure to this highly carcinogenic compound. This prevalence is notably lower than the 75–94% reported in a large longitudinal cohort in rural Nepal [11]. Methodological differences are unlikely to fully explain this contrast. Although the Nepal study employed HPLC with fluorescence detection and reported an LOD of 0.4 pg AFB_1_-lysine/mg albumin, this corresponds to approximately 0.016 ng/mL assuming typical serum albumin concentrations and is therefore comparable to the LOD of 0.015 ng/mL (LOQ 0.04 ng/mL) used in our LC–MS/MS-based method following enzymatic digestion and solid-phase extraction. The lower prevalence observed in our cohort therefore more likely reflects differences in exposure patterns. In the Nepalese cohort, frequent maize and groundnut consumption were strong predictors of higher AFB_1_-lysine levels [12], whereas maize consumption is uncommon in our study population whose main staple is rice, and groundnut consumption was reported by fewer than 10% of pregnant women [52], compared with over 30% in Nepal [12]. Differences in post-harvest storage conditions and fungal contamination profiles of staple foods may further contribute to these contrasting exposure burdens. In our study area, we previously observed limited farmer knowledge regarding the timing, causes, and preventive practices related to mold contamination of crops [53], suggesting that post-harvest vulnerability remains an important local risk factor; similar challenges may also exist in comparable rural agricultural settings such as Nepal. Seasonal variation may also be relevant, as the Nepal study reported higher aflatoxin levels during winter months, while our blood samples were collected largely during autumn (sharat) and late autumn (Hemanta) months.

Despite the low detection frequency, the public health significance of even infrequent AFB_1_-lysine detection in children should not be understated: prior evidence linking AFB_1_-lysine to impaired linear growth [11] and the absence of any safe threshold for this genotoxic carcinogen [21, 35] mean that even sporadic detectable exposure during early childhood, a period of heightened vulnerability, warrants continued surveillance. The presence of AFB_1_-lysine in our cohort, therefore, is concerning: even low-level chronic exposure to aflatoxin B1 is associated with increased hepatocellular carcinoma risk [35], and the growth-related findings from comparable settings suggest potential developmental consequences. Together, these findings suggest that aflatoxin exposure is persistent in this population and underscore the importance of blood-based biomarkers for capturing exposure that may be underestimated by urine biomonitoring alone.

Dietary exposure assessment based on blood-derived PDIs revealed substantially higher OTA and CIT health risk among children compared with women. For OTA, nearly all children exceeded the health concern thresholds (margins of exposure below the critical value of 10,000), regardless of the toxicokinetic model applied. In addition, over one-quarter of children exceeded the tolerable daily intake for citrinin. These findings are concerning, as early life represents a critical window of vulnerability, during which toxic exposures may have disproportionate effects on growth, immune function, and long-term health [59]. Higher health risk among young children likely reflects a combination of children's lower body weight with shared household diets, and potentially higher intake of contaminated foods relative to body mass. Complementary foods in rural Bangladesh are commonly home-prepared from staple grains (e.g., rice-based porridge and Khichuri, a traditional dish of cooked rice and pulses) using storage and preparation practices that may differ from those used for adult meals, and may be more vulnerable to mold contamination given limited refrigeration and variable hygiene practices during preparation and storage [61]. The results align with the concept of the first 1000 days as a sensitive period and underscore the importance of including children in biomonitoring and risk assessment efforts in low- and middle-income settings.

Taken together, our findings indicate that OTA and related compounds, including 2′R-OTA, remain important mycotoxin-related public health concerns that warrant prioritization for further attention in this rural Bangladeshi population, with CIT contributing additional exposure burden, particularly among children. Importantly, the present study, to our knowledge, is the first in Bangladesh to report the blood-based biomarker AFB_1_-lysine, revealing that nearly one-quarter of women have measurable exposure to AFB_1_, a potent human carcinogen, and highlighting a previously underrecognized exposure pathway in this setting. The observed multi-mycotoxin exposure profile is likely driven by a combination of dietary and environmental factors, including reliance on staple-based diets and conditions that may favor fungal contamination along the food production and storage chain. In line with this, a previous survey among the same study population showed limited awareness of good agricultural and storage practices for mitigating fungal growth and mold contamination [53], suggesting that gaps in food handling knowledge may contribute to sustained exposure risk.

Overall, these results underscore the need for integrated preventive strategies targeting post-harvest handling, storage conditions, and dietary diversification. In addition, the widespread co-exposure to multiple mycotoxins suggests that regulatory and public health frameworks focusing on single mycotoxins may substantially underestimate real-world risks.

A strength of this study is the use of blood biomarkers, including protein adducts, which allowed us to assess longer-term exposure and to extend and validate findings from earlier urine-based analyses in the same cohort. A further strength is the inclusion of both women and children which provides insight into shared exposure sources and age-related vulnerability.

Several limitations should be considered. First, regarding the representativeness of the analytic sample: participants were included based on being part of sub-cohorts of pregnant women or young children (aged 6 to 24 months) and on the availability of residual blood volume following testing as part of the trial, a criterion plausibly unrelated to mycotoxin exposure but not formally verified to be independent of it. While we have no specific reason to expect selection bias with respect to mycotoxin exposure status, we cannot rule it out, and the prevalence and intake estimates presented here should be interpreted as describing this analytic sample rather than the full FAARM cohort or the general population of rural Bangladesh. The study cohort comprises children drawn from two nested FAARM sub-studies with partially overlapping but distinct enrollment criteria — MEMAPO, which followed pregnant women (and subsequently their children) from early pregnancy, and FHEED, which assessed a food hygiene intervention among children below 24 months of age. Relatedly, the dyad-level analysis above is restricted to the subset of children with a linked maternal record with available whole blood biomarker data (87 of 284 children, 31%). Most unmatched children were enrolled through FHEED without a mother who also participated in MEMAPO, and so lack a linked maternal blood sample. The 87 available dyads may therefore not be representative of the full child sample, and dyad-level findings should be treated as exploratory rather than a representative estimate of within-household correlation across the cohort.

Second, our exposure and risk estimates carry several layers of uncertainty stemming from the modeling approach itself. Our risk characterization followed a deterministic approach, using point estimates of toxicokinetic parameters (plasma clearance, bioavailability) and fixed substitution values for left-censored data, rather than propagating uncertainty and inter-individual variability through probabilistic modeling. The reported proportions of participants exceeding health-based guidance values are therefore best interpreted as approximate, population-level screening indicators rather than precise prevalence estimates. The probable daily intake (PDI) calculation itself further attributes all measured systemic mycotoxin burden to dietary intake. To the extent that non-dietary routes (e.g., inhalation, dermal contact) contribute to measured blood concentrations, our PDI estimates would overestimate dietary-specific exposure specifically, although total systemic exposure estimates would remain valid. Additional uncertainty arises from the toxicokinetic models used for OTA: the approximately 2.3-fold difference in PDI between Model 1 and Model 2 reflects genuine ambiguity in the underlying human clearance data [73, 74] rather than a methodological error, and we retain both models here to bracket this uncertainty rather than to imply that the higher Model 2 estimates should be read in isolation as a precise exposure figure. Future studies would benefit from probabilistic approaches, such as Monte Carlo simulation, to formally propagate uncertainty in toxicokinetic parameters, analytical measurement, and left-censoring treatment into final exposure and risk estimates.

Third, several limitations are specific to particular subgroups or biomarkers. The toxicokinetic parameters used for OTA and CIT intake estimation were derived from adult studies and may not accurately reflect pediatric clearance dynamics. As children exhibit age-dependent maturation of elimination pathways, estimated pediatric PDIs should be interpreted with particular caution. Toxicological reference values are also unavailable for several emerging mycotoxins and OTA degradation products, such as 2′R-OTA, precluding formal risk assessment for these compounds. For AFB_1_-lysine, the high proportion of left-censored observations, particularly in children, limited robust distributional modeling. The lower-bound substitution used for correlation analyses may underestimate true variability and should be interpreted cautiously.

Finally, although co-exposure to multiple mycotoxins was nearly universal in this cohort, risk characterization was performed for OTA and CIT individually, because established cumulative assessment frameworks are currently lacking for this mycotoxin combination. This approach does not account for possible additive or synergistic toxic effects and may therefore underestimate overall health risk from chronic mycotoxin mixtures. In contrast to the deterministic and toxicokinetic sources of uncertainty discussed above, which generally risk over-estimating exposure or risk, in this respect our approach may instead underestimate true risk. The precautionary application of the Margin of Exposure approach to OTA, based on its classification as a substance with potential genotoxic carcinogenicity, may itself represent a conservative framework that overstates risk relative to a non-genotoxic, threshold-based assessment, if OTA's carcinogenic mechanism does not, in fact, involve direct genotoxic action (see Health risk characterization, Methods). Future work should consider cumulative risk assessment approaches, such as hazard index-based or relative potency factor frameworks, as sufficient toxicological data for mycotoxin mixtures become available.

## Conclusions

This study provides consistent evidence of chronic, multi-mycotoxin exposure among women and children in rural Bangladesh, dominated by OTA, its heat-induced diastereomer 2′R-OTA, and CIT. Children experience a disproportionately higher exposure burden and associated health risk for OTA and CIT specifically. In addition, this study provides the first blood-based evidence of AFB_1_ exposure in Bangladesh, with nearly one-quarter of women showing measurable AFB_1_-lysine adduct levels — a previously underrecognized exposure pathway that, despite lower detection frequency in children, warrants continued surveillance given the absence of a safe threshold for this genotoxic carcinogen. This study provides consistent evidence of chronic, multi-mycotoxin exposure among women and children in rural Bangladesh, dominated by OTA, its heat-induced diastereomer 2′R-OTA, and CIT. Children experience a disproportionately higher exposure burden and associated health risk for OTA and CIT specifically. In addition, this study provides the first blood-based evidence of AFB_1_ exposure in Bangladesh, with nearly one-quarter of women showing measurable AFB_1_-lysine adduct levels — a previously underrecognized exposure pathway that, despite lower detection frequency in children, warrants continued surveillance given the absence of a safe threshold for this genotoxic carcinogen.

## Supplementary Information

Supplementary Material 1.

## Data Availability

Data Availability Statement: The data presented in this study are available upon reasonable request from the corresponding author. The data are not publicly available due to privacy restrictions.
