# Supplementary material for: Chronic exposure to aflatoxin B1, ochratoxin A, and citrinin in women and children and associated health risk characterization: blood biomonitoring evidence from rural Bangladesh
Source: Environ Health. 2026 Sep 2;25:75. doi: 10.1186/s12940-026-01330-7 (PMC13563859; doi:10.1186/s12940-026-01330-7)
Supplement: Supplementary file 1 [file 12940_2026_1330_MOESM1_ESM.docx]

**SUPPLEMENTARY**

**METHODS S1**

**S1.1 — Derivation of the ochratoxin A (OTA) intake equation**

As described in the main text, two toxicokinetic models were used to estimate OTA intake, both derived from the Klaassen equation describing the relationship between total body clearance and steady-state plasma concentration (Klaassen 1986):

k0 = (Clp × Cp) / A

where k0 is the daily intake of OTA (ng OTA/kg body weight/day), Clp is the plasma clearance (mL/kg body weight/day), Cp is the plasma concentration of OTA (ng/mL), and A is the bioavailability of OTA (fraction absorbed).

**Model 1.** This model is based on a human toxicokinetic study of OTA described in a doctoral dissertation by Studer-Rohr, who compared renal and plasma clearance of OTA and concluded that non-renal elimination pathways were negligible (Studer-Rohr 1995). Assuming a standard adult body weight of 70 kg, a plasma clearance of 0.048 mL/min, a conversion factor of 1440 minutes/day, and a bioavailability of 0.5, the equation reduces to:

k0 = 1.97 × Cp (Model 1)

**Model 2.** The same human kinetic study was subsequently republished with recalculated toxicokinetic parameters, yielding a higher renal clearance estimate of 0.1099 mL/min (Studer-Rohr et al. 2000). Applying this revised clearance value under the same assumptions as above gives an alternative simplified coefficient:

k0 = 4.52 × Cp (Model 2)

Both models are applied in the main analysis, following Warensjö Lemming et al. (2020), to allow comparison and to account for uncertainty in OTA toxicokinetic parameters arising from the revised clearance estimate. Unlike the standard application of these models in the literature, individual body weights of participants (rather than a fixed 70 kg) were incorporated directly into the underlying equation in the main analysis, given the substantially lower average body weight in this study population (about 49 kg for women and about 8 kg for children).

**S1.2 — Derivation of the citrinin (CIT) intake equation**

As described in the main text, citrinin intake was estimated via the Klaassen equation:

K0 = (Clp × Cp / A) × τ

Plasma clearance (Clp) was calculated as:

Clp = (D/AUC) × f

where D is the administered dose, AUC is the area under the plasma concentration-time curve from an oral dosing study, and f is the fraction absorbed (oral bioavailability). Because oral bioavailability is lower than intravenous bioavailability, this correction for f is necessary; f was estimated from the cumulative urinary excretion of citrinin and its metabolite, dihydrocitrinone.

Substituting this expression for Clp into the Klaassen equation gives:

K0 = [((D/AUC) × f) × Cp / f] × τ

The bioavailability term (f) cancels:

K0 = (D/AUC) × Cp × τ

This cancellation is methodologically useful because it removes the need to independently estimate oral bioavailability (f) from urinary excretion data, which would otherwise introduce additional uncertainty into the intake estimate. Using a dosing interval (τ) of 1440 minutes (24 hours) and normalizing by body weight, the final equation used in the main analysis is:

K0 = (11.5 / body weight) × Cp × 1440

(Degen et al. 2018)

**Table S1**: **Mass spectrometer parameters for multiple reaction monitoring detection used for the analysis of mycotoxins in blood samples.**

| Analyte | Q1 mass [*m/z*] | Q3 mass [*m/z*] | EP [V] | CE [eV] | CXP [V] |
| --- | --- | --- | --- | --- | --- |
| AFB_1_ | 313.1 | 285.0 | 10 | 34 | 20 |
|  | 313.1 | 241.0 | 10 | 54 | 17 |
|  | 313.1 | 269.0 | 10 | 43 | 19 |
|  | 313.1 | 213.0 | 10 | 62 | 14 |
| AFB_2_ | 315.1 | 287.1 | 10 | 37 | 20 |
|  | 315.1 | 259.0 | 10 | 41 | 20 |
| AFG_1_ | 329.1 | 243.0 | 10 | 39 | 19 |
|  | 329.1 | 311.0 | 10 | 31 | 19 |
|  | 329.1 | 199.0 | 10 | 68 | 17 |
|  | 329.1 | 215.1 | 10 | 46 | 16 |
| AFG_2_ | 331.2 | 303.1 | 10 | 36 | 17 |
|  | 331.2 | 245.1 | 10 | 41 | 17 |
| AFM_1_ | 329.1 | 273.0 | 10 | 34 | 15 |
|  | 329.1 | 229.0 | 10 | 54 | 15 |
| ALT | 293.1 | 197.0 | 10 | 39 | 12 |
|  | 293.1 | 152.0 | 10 | 62 | 12 |
| AME | 271.1 | 213.0 | -10 | -45 | -20 |
|  | 271.1 | 211.0 | -10 | -50 | -20 |
|  | 271.1 | 256.0 | -10 | -32 | -15 |
| AOH | 257 | 213.1 | -10 | -34 | -15 |
|  | 257 | 215.0 | -10 | -38 | -15 |
|  | 257 | 212.0 | -10 | -39 | -16 |
| Bea | 784.4 | 244.1 | 10 | 37 | 25 |
|  | 784.4 | 262.2 | 10 | 36 | 25 |
| CIT | **251.1** | **91.0** | **10** | **56** | **13** |
|  | 251.1 | 115.0 | 10 | 68 | 13 |
| DH-CIT | **267.1** | **231.0** | **10** | **35** | **19** |
|  | 267.1 | 203.0 | 10 | 40 | 14 |
| DON | 297.1 | 231.0 | 10 | 17 | 11 |
|  | 297.1 | 219.0 | 10 | 17 | 11 |
|  | 297.1 | 159.0 | 10 | 26 | 9 |
| DON-3-GlcA | 471.2 | 59.0 | -10 | -72 | -7 |
|  | 471.2 | 265.1 | -10 | -38 | -15 |
|  | 471.2 | 175.0 | -10 | -38 | -15 |
| EnA | 704.4 | 350.1 | 10 | 68 | 25 |
|  | 704.4 | 232.0 | 10 | 73 | 25 |
| EnA_1_ | 668.4 | 210.1 | 10 | 33 | 25 |
|  | 668.4 | 228.3 | 10 | 35 | 25 |
| EnB | 640.4 [M+H]^+^ | 196.2 | 10 | 32 | 11 |
|  | 662.4 [M+Na]^+^ | 336.3 | 10 | 63 | 15 |
|  | 657.4 [M+NH_4_]^+^ | 196.1 | 10 | 43 | 19 |
| EnB_1_ | 654.4 | 214.2 | 10 | 35 | 25 |
|  | 654.4 | 196.1 | 10 | 52 | 25 |
| FB_1_ | 722.4 | 334.3 | 10 | 56 | 24 |
|  | 722.4 | 352.3 | 10 | 51 | 19 |
| OTA/2’*R*-OTA | **404.1** | **239.0** | **10** | **36** | **14** |
|  | 404.1 | 221.0 | 10 | 50 | 16 |
| OTα | 257 | 193.0 | 10 | 37 | 23 |
|  | 257 | 102.0 | 10 | 54 | 12 |
| 10-OH-OTA | 420.1 | 255.0 | 10 | 36 | 13 |
|  | 420.1 | 374.0 | 10 | 21 | 21 |
|  | 420.1 | 237.0 | 10 | 50 | 16 |
| HT-2 | 447.2 [M+Na]^+^ | 345.0 | 10 | 25 | 11 |
|  | 447.2 [M+Na]^+^ | 285.0 | 10 | 30 | 11 |
|  | 447.2 [M+Na]^+^ | 267.1 | 10 | 30 | 14 |
|  | 442.4 [M+NH_4_]^+^ | 215.2 | 10 | 18 | 18 |
| HT-2-4-GlcA | 623.2 [M+Na]^+^ | 345.101 | 10 | 36 | 15 |
|  | 623.2 [M+Na]^+^ | 285.101 | 10 | 42 | 15 |
| T-2 | 489.2 [M+Na]^+^ | 387.201 | 10 | 29 | 13 |
|  | 489.2 [M+Na]^+^ | 327.201 | 10 | 34 | 13 |
| ZAN | 319.1 | 205.001 | -10 | -31 | -13 |
|  | 319.1 | 161.001 | -10 | -37 | -13 |
| ZEN | 317.2 | 175.001 | -10 | -34 | -12 |
|  | 317.2 | 130.989 | -10 | -40 | -12 |
|  | 317.2 | 187.043 | -10 | -34 | -14 |
| d5-OTA/ d5-2’*R*-OTA | 409.1 | 239.0 | 10 | 36 | 14 |
|  | 409.1 | 221.0 | 10 | 50 | 16 |
| ^13^C_3_-CIT | 254.1 | 94.0 | 10 | 56 | 13 |
|  | 254.1 | 118.0 | 10 | 68 | 13 |
| ^13^C_3_-DH-CIT | 270.1 | 206.0 | 10 | 35 | 19 |
|  | 270.1 | 234.0 | 10 | 40 | 14 |

**Note:** Quantifier transitions are indicated in bold. EP: entrance potential; CE: collision energy; CXP: collision cell exit potential. Analytes comprise a panel of mycotoxins and related metabolites, including aflatoxins (AFB₁, AFB₂, AFG₁, AFG₂, AFM₁), Alternaria toxins (AOH, AME, ALT), enniatins (EnA, EnA1, EnB, EnB1), fumonisins (FB₁), trichothecenes (DON, HT-2, T-2 and their glucuronide conjugates), zearalenone and derivatives (ZEN, ZAN), ochratoxins (OTA, 2’R-OTA, OTα, 10-OH-OTA), citrinin and metabolites (CIT, DH-CIT), beauvericin (Bea), as well as isotopically labelled internal standards (e.g., d₅-OTA, ¹³C₃-CIT, ¹³C₃-DH-CIT). Q1 and Q3 denote the mass-to-charge ratios (m/z) of the precursor ion and product (fragment) ion, respectively, monitored in multiple reaction monitoring (MRM) mode using tandem mass spectrometry (LC-MS/MS).

**Table S2:** Sp**earman correlations between blood-based mycotoxin biomarkers in mothers and children, rural Habiganj district, Bangladesh.**

| **Biomarker pair** | **Sample size (n)** | **Spearman’s ρ** | **p-value** |
| --- | --- | --- | --- |
| CIT – DH-CIT | 712 | 0.53 | <0.001 |
| OTA – CIT | 712 | 0.28 | <0.001 |
| AFB1-lysine – OTA | 571 | 0.24 | <0.001 |

CIT: citrinin; DH-CIT: dihydrocitrinone (a major metabolite of citrinin); OTA: ochratoxin A; AFB₁-lysine: aflatoxin B₁–lysine adducts, a protein-bound biomarker of aflatoxin B₁ exposure. Spearman’s ρ (rho) denotes the non-parametric rank correlation coefficient, and p-values indicate the statistical significance of the observed correlations.

**Table S3. Spearman correlation of plasma ochratoxin A (OTA) and citrinin (CIT) concentrations between mothers and their children, rural Habiganj district, Bangladesh.**

| **Mycotoxin** | **n (dyads)** | **Spearman's ρ** | **p-value** |
| --- | --- | --- | --- |
| OTA | 85 | 0.468 | <0.001 |
| CIT | 68 | 0.014 | 0.913 |

*Dyads restricted to mother-child pairs with both plasma OTA or CIT concentrations available (n = 87 dyads identified out of 284 children analyzed; n differs between OTA and CIT due to left-censored or missing values for individual biomarkers within some dyads).*

**Table S4: Detection of enniatin B and beauvericin in human whole blood samples from rural Habiganj district, Bangladesh**

| **Mycotoxin biomarker** | **Instrumental limits for biomarkers (ng/ml)** | **Analyzed samples** | **Total detected** | **Detected, n (% ≥LOD)** | **Quantified, n (% ≥LOQ)** |
| --- | --- | --- | --- | --- | --- |
|  | LOD |  |  |  |  |
| EnB | 0.001 |  |  |  |  |
| women |  | 428 | 396 | 269 (92.5) | 127 (29.7) |
| children |  | 284 | 257 | 140 (90.5) | 117 (41.2) |
| All |  | **712** | **653** | **409 (91.7)** | 244 (34.3) |
| BEA | 0.013 |  |  |  |  |
| women |  | 428 | 29 | 22 (6.8) | 7 (1.6) |
| children |  | 284 | 13 | 10 (4.6) | 3 (1.1) |
| All |  | **712** | **42** | **32 (5.9)** | 10 (1.4) |

EnB: Enniatin B; BEA: Beauvericin

|  | Frequency |
| --- | --- |
| tOTA_tCIT_EnB | 560 |
| tOTA_EnB | 51 |
| tOTA_tCIT | 50 |
| tOTA_tCIT_EnB_BEA | 38 |
| tOTA | 8 |
| tOTA_EnB_BEA | 4 |
| tOTA_tCIT_BEA | 1 |

*t*CIT – total CIT (CIT + DH-CH); *t*OTA – total OTA (OTA + 2’R-OTA)

**Figure S1: Distribution of co-exposure patterns based on combinations of detected plasma mycotoxin biomarkers from women and children in rural Habiganj, Bangladesh (n=712).** The pie chart shows the frequency and proportion of participants with different combinations of detected mycotoxin groups, including total ochratoxin A (tOTA; sum of ochratoxin A [OTA] and its isomer 2′R-ochratoxin A), total citrinin (tCIT; sum of citrinin [CIT] and dihydrocitrinone [DH-CIT]), enniatin B (EnB), and beauvericin (BEA). Nearly all (99%, all except the blue section) of participants were concurrently exposed to multiple mycotoxins, most commonly the combination of tOTA, tCIT, and EnB (79%, yellow section).

**Figure S2: Scatterplots of pairwise correlations between selected blood-based mycotoxin biomarkers.** Scatterplots show pairwise relationships between the mycotoxin biomarkers for which Spearman correlation coefficients are reported in Table S2: CIT and DH-CIT, OTA and CIT, and AFB1-lysine and OTA. The red line represents the fitted linear regression line for visualisation of trends. Biomarker concentrations are shown on the original scale. CIT = citrinin; DH-CIT = dihydrocitrinone (citrinin metabolite); OTA = ochratoxin A; AFB1-lysine = aflatoxin B1–lysine albumin adduct.
